# An Alternative Technique for Surgical Management of Poststernotomy Osteomyelitis and Reconstruction of the Sternal Defect

**DOI:** 10.1155/2013/451594

**Published:** 2013-03-03

**Authors:** Petros Konofaos, Eleftherios Spartalis, Grigorios Karagkiouzis, Christos Kampolis, Periklis Tomos

**Affiliations:** ^1^Department of Plastic, Reconstructive and Craniofacial Surgery, Health Science Center, University of Tennessee, Memphis, TN 38163, USA; ^2^Department of Plastic Surgery, Medical School, Athens University, 106 76 Athens, Greece; ^3^Second Department of Propaedeutic Surgery, Medical School, Athens University, 106 76 Athens, Greece

## Abstract

*Introduction.* Sternal osteomyelitis with or without mediastinal infection is a severe and rare complication of median sternotomy. In this paper, an alternative technique for the reconstruction of sternal defects with the use of bilateral pectoralis major pedicled muscle flaps is presented. *Case presentation.* A 70-year-old man with the diagnosis of poststernotomy osteomyelitis underwent reconstruction of his sternal defect with the use of bilateral pectoralis major muscle flaps. The patient had an uneventful recovery, and the physical examination revealed a normal range of motion for both upper limbs and sternal stability. *Conclusion.* The proposed technique incorporates a simple mobilization of the two pectoralis major muscles to be used as flaps to fill the sternal defect without the need for humeral detachment or a second cutaneous incision. Using this technique, a muscular implant is made that seals the dead space, which has no tension due to the presence of a second layer. Postoperative results are excellent, not only regarding infection and functionality but also from an aesthetic point of view.

## 1. Introduction

Poststernotomy infection due to coronary artery bypass grafting represents one of the greatest challenges for the reconstructive surgeon. Its incidence is ranged between 1% and 4% [1, 2]. Mainstay of the reconstruction of sternal defects is to provide long term and stable coverage of thoracic viscera without marked patient morbidity. Many authors have described the use of other muscles as flaps [3, 4]. In this paper, we evaluate the postoperative results of the use of the bilateral pectoralis major pedicled muscle flap with the use of an alternative technique for reconstruction of sternal defects.

## 2. Case Presentation

**Figure 1:**
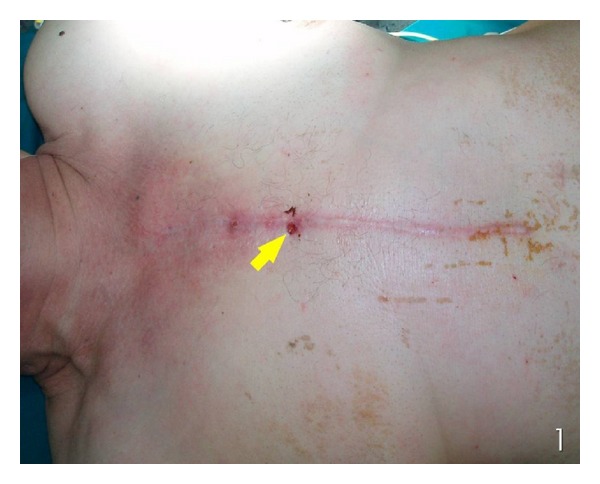
Sternocutaneous fistula (arrow).

**Figure 2:**
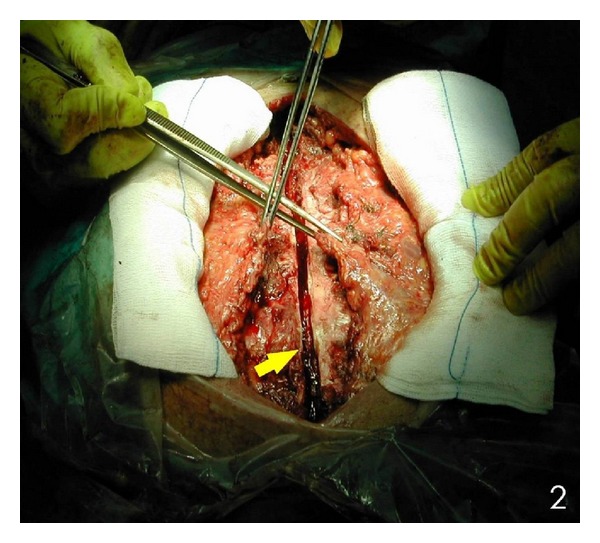
The defect after the V sternectomy (arrow). The raising of the pectoralis major muscle flaps (forceps).

**Figure 3:**
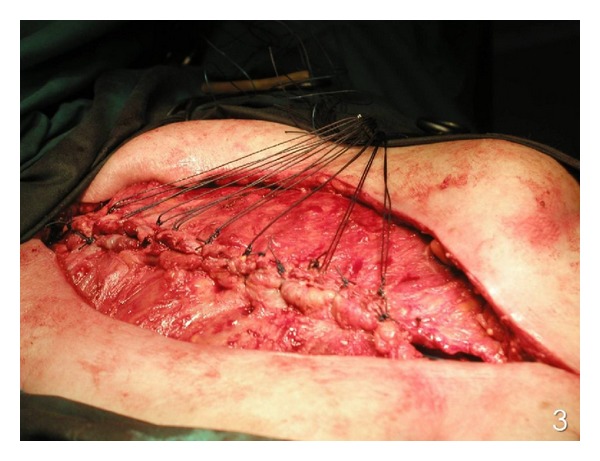
Final result after suturing the medial ends of both flaps.

**Figure 4:**
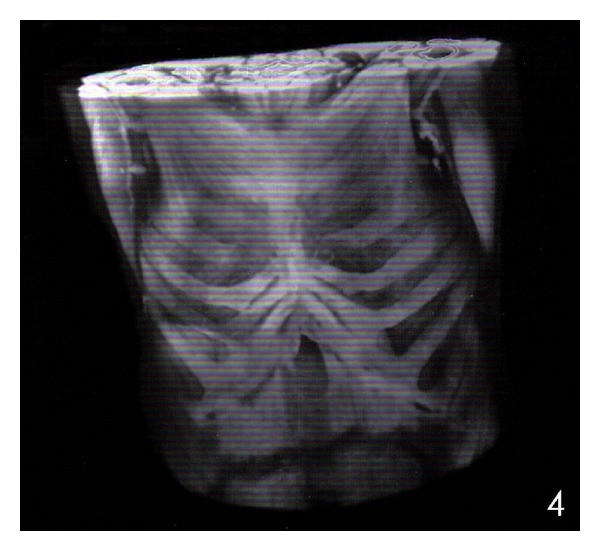
Postoperative chest wall MRI.

A 70-year-old man, diabetic, with ejection fraction of about 20%, was referred at the Second Department of Propaedeutic Surgery of Athens University with a chest pain and a sternocutaneous fistulous tract discharging pus (Figure 1). Seven weeks earlier, he had undergone a redo coronary artery bypass via median sternotomy. The routine laboratory tests were within normal limits, except an elevated white blood cell count (18 × 10^3^/*μ*L) and high *CRP* level (135 mg/L). Arterial blood gas analysis on admission day was  PO_2_: 86 mmHg, PCO_2_: 39 mmHg, pH: 7.4, SPO_2_: 96%, and HCO_3_^−^: 23.6 mmol/L. The diagnosis of poststernotomy sternal infection was set based on the patient's medical history and clinical examination. The culture of the pus revealed *methicillin resistant Staphylococcus aureus (MRSA).* This infection was treated with administration of vancomycin for 6 weeks on the basis of microbiologic susceptibility. The patient underwent reconstruction of his sternal defect with the use of an alternative technique proposed by Tomos et al. [5]. The procedure was started with a V-shape partial sternectomy of the midline (Figure 2). The thoracic skin was undermined over both pectoralis major muscles from the margin of the defect as wide as needed. The pectoralis major muscle (flap 1), in which its internal mammary artery had been used for coronary artery bypass, was mobilized until the level of the anterior axillary line from its chest wall insertion. The contralateral pectoralis major muscle (flap 2) was mobilized until the middle of the distance between the margin of the defect and the anterior axillary line from its chest wall insertion. Intercostal perforators towards the skin were ligated carefully without damaging their intramuscular flow. The clavicular insertions and the humeral insertions of both pectoralis flaps were left intact. Moreover, the vascular system of the perforators of these two flaps was remaining intact. The medial ends of both the pedicled pectoralis major muscle flaps were placed into the dead space, with the flap 1 to locate upwards and the flap 2 to locate downwards. A single row of interrupted, half-buried, vertical mattress stitches were placed, starting from 2 cm laterally to the medial end of the flap 1 in an anterior-posterior direction, then proceeding to the flap 2, 2 cm laterally to its medial end in an anterior-posterior-anterior direction and then back again but this time 1 cm caudal in an anterior-posterior-anterior direction. Finally, the wire passes from the posterior to anterior surface of the flap 1, which created an overlap of the flaps so that the flap 2 was buried in the V scraped area, and the flap 1 was overlapping (Figure 3). All these sutures passed right through the muscles. The patient had an uneventful recovery and was discharged from the hospital on the 12th postoperative day. Postoperative MRI (Figure 4), 6 months after surgery, did not show any pathological finding, and physical examination revealed a normal range of motion for both upper limbs and sternal stability.

## 3. Discussion

The goals of the treatment of postoperative sternal osteomyelitis and mediastinitis are to resolve the infectious process in the shortest time possible, ensure sternal stability, and provide the best possible cosmetic results. Many innovative techniques have been proposed by many authors for the reconstruction of sternal defects with the use of pectoralis major flaps. Jurkiewicz and others [3, 6] transposed the pectoralis major into the mediastinum based on either the thoracoacromial pedicle or as a turnover flap based on perforators of the internal mammary artery. Nahai et al. [7] introduced a modification of the turnover pectoralis major flap by dividing the muscle medial to the thoracoacromial pedicle and using only the medial two thirds of the pectoralis major for definitive coverage of the mediastinal wound. Tobin [8] suggested the splitting of the pectoralis major muscle into sternocostal, external, and clavicular segments and the preserving of the thoracoacromial pedicle. Morain et al. [9] suggested the splitting of the pectoralis major muscle into segments but leaving some of the segments intact to preserve muscle function for smaller defects requiring only a small portion of muscle. The main disadvantage of all the above-mentioned techniques is that, many times, the use of the contralateral pectoralis muscle flap is imperative for the closure of the sternal defect. The use of both the pectoralis major advancement pedicled muscle flaps for the reconstruction of a sternal defect is associated with the avoidance of more distant donor sites particularly that of the abdomen, which is, in our opinion, very important in decreasing morbidity and hospital stay. The main advantage of this presenting technique is that the pedicles of both the muscle flaps are remain intact. Flaps blood supply is based on perforating muscle branches of the internal mammary artery, on the lateral thoracic artery and on the thoracoacromial artery for the flap 2, whereas for the flap 1, it is based on the lateral thoracic artery and on the thoracoacromial artery. In contrast to other techniques [9] which are isolated and cauterized the anterior perforating arteries when they created myocutaneous flaps with the pectoralis muscles, our technique succeeds in creating two pedicled muscle flaps with intact to their vascular pedicles, and this fact is associated with decreased morbidity and reinfection rate. Moreover, the range of motion of the shoulders joints remains intact because the clavicular and humeral insertions of both the pectoralis muscles are remain intact. There is a decrease in the range of motion of both arms because the percentage of the pectoralis muscle flaps used for covering the defect is limited in terms of the flaps size. Another advantage of this technique is the stability of the sternal area because both the medial ends of the pectoralis major used for covering the sternal defect. Moreover, due to the intact vascular system of both flaps, the application to the sternal defect of a well-vascularized tissue offers an effective way for the opposition of the inflammation at the defect area.

It should also be noted that, with our technique, there are no significant donor site deformities, unlike many other techniques, that use flaps of the pectoralis muscle and especially the turnover flap. There is no depression at the donor site, and there is no distortion of the breast. According to the literature, the use of flap transposition for the reconstruction of sternal defects is characterized by an increase probability of significant short and long-term complications [10, 11]. Using this alternative technique, these complications seem to be reduced because of the preservation of the vascular supply of the flaps during the operation and the way the flaps cover the defect which creates the appropriate sternal stability. The selection of the appropriate flap is based on the one hand at the size and depth of the sternal defect and, on the other hand, at the experience of the operating surgeon. We believe that this technique may offer an excellent and safe reconstruction solution in many cases of postoperative sternal infections.

## 4. Conclusions

The proposed technique incorporates a simple mobilization of the two pectoralis major muscles for use as flaps to fill the sternal defect without the need for humeral detachment or a second cutaneous incision. Using this technique, a muscular implant is made that seals the dead space, which has no tension due to the presence of a second layer. Postoperative results are excellent, not only regarding infection and functionality but also from an aesthetic point of view.

## References

[1] Grossi EA, Culliford AT, Krieger KH (1985). A survey of 77 major infectious complications of median sternotomy: a review of 7,949 consecutive operative procedures. *Annals of Thoracic Surgery*.

[2] The Parisian Mediastinitis Study Group (1996). Risk factors for deep sternal wound infections after sternotomy: a prospective, multicenter study. *The Journal of Thoracic and Cardiovascular Surgery*.

[3] Jurkiewicz MJ, Bostwick J, Hester TR (1980). Infected median sternotomy wound. Successful treatment by muscle flaps. *Annals of Surgery*.

[4] Hugo NE, Sultan MR, Ascherman JA, Patsis MC, Smith CR, Rose EA (1994). Single-stage management of 74 consecutive sternal wound complications with pectoralis major myocutaneous advancement flaps. *Plastic and Reconstructive Surgery*.

[5] Tomos P, Lachanas E, Michail PO, Kostakis A (2006). Alternative bi-pectoral muscle flaps for postoperative sternotomy mediastinitis. *Annals of Thoracic Surgery*.

[6] Brown RG, Fleming WH, Jurkiewicz MJ (1977). An island flap of the pectoralis major muscle. *British Journal of Plastic Surgery*.

[7] Nahai F, Morales L, Bone DK, Bostwick J (1982). Pectoralis major muscle turnover flaps for closure of the infected sternotomy wound with preservation of form and function. *Plastic and Reconstructive Surgery*.

[8] Tobin GR (1990). Segmentally split pectoral girdle muscle flaps for chest-wall and intrathoracic reconstruction. *Clinics in Plastic Surgery*.

[9] Morain WD, Colen LB, Hutchings JC (1985). The segmental pectoralis major muscle flap: a function-preserving procedure. *Plastic and Reconstructive Surgery*.

[10] Pairolero PC, Arnold PG, Harris JB (1991). Long-term results of pectoralis major muscle transposition for infected sternotomy wounds. *Annals of Surgery*.

[11] Ringelman PR, Kolk CAV, Cameron D, Baumgartner WA, Manson PN, Nahai F (1994). Long-term results of flap reconstruction in median sternotomy wound infections. *Plastic and Reconstructive Surgery*.

